# Effects of autophagy modulators tamoxifen and chloroquine on the expression profiles of long non-coding RNAs in MIAMI cells exposed to IFNγ

**DOI:** 10.1371/journal.pone.0266179

**Published:** 2022-04-21

**Authors:** Rajkaran Banga, Veerkaran Banga, Amr Eltalla, Lauren Shahin, Sonam Parag, Maha Naim, Easha Iyer, Neha Kumrah, Brian Zacharias, Lubov Nathanson, Vladimir Beljanski

**Affiliations:** 1 Dr Kiran C. Patel College of Allopathic Medicine, Nova Southeastern University, Davie, Florida; 2 Dr Kiran C. Patel College of Osteopathic Medicine, Nova Southeastern University, Davie, Florida; 3 Department of Biological Sciences, Halmos College of Arts and Sciences, Nova Southeastern University, Fort Lauderdale, Davie, Florida; 4 Institute for Neuroimmune Medicine, Dr Kiran C. Patel College of Osteopathic Medicine, Nova Southeastern University, Davie, Florida; 5 Cell Therapy Institute, Dr Kiran C. Patel College of Allopathic Medicine, Nova Southeastern University, Davie, Florida; Northwest University, UNITED STATES

## Abstract

Mesenchymal stromal cells (MSCs) can be utilized clinically for treatment of conditions that result from excessive inflammation. In a pro-inflammatory environment, MSCs adopt an anti-inflammatory phenotype resulting in immunomodulation. A sub-type of MSCs referred to as “marrow-isolated adult multilineage inducible” (MIAMI) cells, which were isolated from bone marrow, were utilized to show that the addition of autophagy modulators, tamoxifen (TX) or chloroquine (CQ), can alter how MIAMI cells respond to IFNγ exposure in vitro resulting in an increased immunoregulatory capacity of the MIAMI cells. Molecularly, it was also shown that TX and CQ each alter both the levels of immunomodulatory genes and microRNAs which target such genes. However, the role of other non-coding RNAs (ncRNAs) such as long non-coding RNAs (lncRNAs) in regulating the response of MSCs to inflammation has been poorly studied. Here, we utilized transcriptomics and data mining to analyze the putative roles of various differentially regulated lncRNAs in MIAMI cells exposed to IFNγ with (or without) TX or CQ. The aim of this study was to investigate how the addition of TX and CQ alters lncRNA levels and evaluate how such changes could alter previously observed TX- and CQ-driven changes to the immunomodulatory properties of MIAMI cells. Data analysis revealed 693 long intergenic non-coding RNAS (lincRNAs), 480 pseudogenes, and 642 antisense RNAs that were differentially regulated with IFNγ, IFNγ+TX and IFNγ+CQ treatments. Further analysis of these RNA species based on the existing literature data revealed 6 antisense RNAs, 2 pseudogenes, and 5 lincRNAs that have the potential to modulate MIAMI cell’s response to IFNγ treatment. Functional analysis of these genomic species based on current literature linking inflammatory response and ncRNAs indicated their potential for regulation of several key pro- and anti-inflammatory responses, including NFκB signaling, cytokine secretion and auto-immune responses. Overall, this work found potential involvement of multiple pro-and anti-inflammatory pathways and molecules in modulating MIAMI cells’ response to inflammation.

## Introduction

Multipotent stem/stromal cells such as mesenchymal stromal/stem cells (MSCs) can be used clinically in the treatment of various conditions typically associated with excessive inflammation [1]. MSCs can be isolated from several adult tissues such as bone marrow, adipose tissue, and the umbilical cord; these cells can adopt an anti-inflammatory phenotype when exposed to pro-inflammatory cytokines such as IFNγ and secrete soluble factors resulting in the induction of an immunomodulatory environment [2]. Thus, in a clinical setting, MSCs are now being explored for the treatment of various conditions associated with excessive inflammation, including graft *vs* host disease (GVHD) and several autoimmune diseases such as ulcerative colitis and rheumatoid arthritis [3]. Nevertheless, several issues that limit the therapeutic use of MSCs have been identified as well. Because MSCs are isolated from various adult tissues and subjects of different ages, they have variable properties, including proliferation and differentiation capacities [1]. Moreover, continuous passaging of MSCs in vitro quickly leads to a replicative senescence and a dramatic decrease in their therapeutic capacity [4, 5]. Additionally, isolation of MSCs from living donors can be associated with risks which include donor morbidity.

Approaches that can potentiate the immunoregulatory properties of MSCs by “enhancing” their anti-inflammatory response in a pro-inflammatory milieu are being evaluated by several laboratories [6–8]. Autophagy, the cellular catabolic stress-response pathway, contributes to many key aspects of such cellular responses, including antigen presentation and cytokine secretion [9]. Previously, we utilized a subpopulation of MSCs termed “MIAMI” (marrow-isolated adult multilineage inducible) to study mRNA expression and associated signaling pathways involved in cellular responses to inflammation in the presence of autophagy modulators [7]. We found that the addition of autophagy stimulator tamoxifen (TX) or autophagy inhibitor chloroquine (CQ) changed both the mRNA and protein levels of key genes regulating anti-inflammatory properties of MSCs. Moreover, TX- and CQ-mediated changes in gene expression predicted the enhancement of MIAMI cells’ immunoregulatory properties due to co-treatments which was confirmed using co-culture functional assays with activated CD4+ T-cells’ in vitro [7]. Interestingly, we also found that co-treatment with either TX or CQ also affected the expression of microRNA (miRNA) targeting immunomodulatory genes. This was an unexpected result because it implied that small molecules that modulate autophagy (which is typically regulated via post-translational mechanisms [10]) also have a capacity to alter gene expression profiles via multiple complementary mechanisms that involve non-coding RNA species.

**Fig 1:**
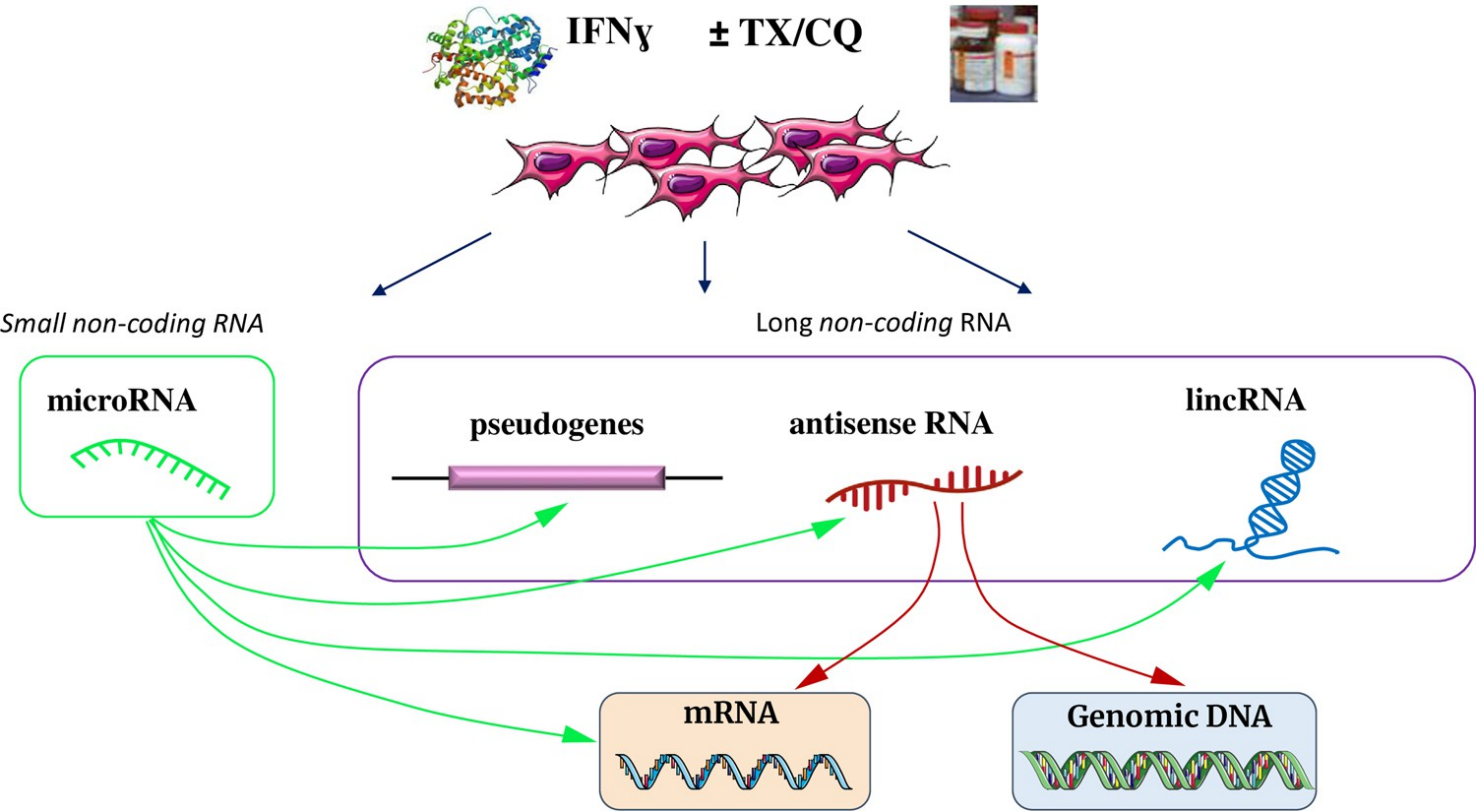
Major interactions between non-coding RNAs, mRNA and DNA. Interactions of these molecular species with proteins are omitted for clarity.

Noncoding RNAs (ncRNAs) can be classified as house-keeping or regulatory molecules, however, they lack protein-coding potential but account for the majority of the transcriptome (~98%); generally, ncRNAs contribute to gene expression regulation (both transcription and post-transcription) and possess additional roles in cellular homeostasis (reviewed in [11]). ncRNAs are comprised of short ncRNAs such as miRNAs, short interfering RNAs and Piwi-associated RNAs which are typically shorter than 30 nucleotides [12]. On the other hand, long non-coding RNAs (lncRNAs) such as pseudogene RNAs, antisense RNAs and the long intergenic ncRNAs (lincRNAs) are typically longer than 200 nucleotides [13]. Unfortunately, the exact physiological function is unknown for the majority of transcribed lncRNAs; furthermore, the role of lncRNAs in regulation of MSCs anti-inflammatory response is currently understudied. Nevertheless, emerging research indicates that the functions of lncRNAs are manifested at transcriptional, post-transcriptional, and post-translational levels by three types of interactions: RNA-RNA, RNA-DNA, and RNA-protein [14]. Thus, mechanisms by which various lncRNAs regulate gene expression involve epigenetic regulation (via genetic imprinting and chromatin remodeling), regulation of transcription (by serving as molecular decoys), post-transcriptional regulation (via splicing and mRNA decay), and, in some cases, translational regulation (**Fig 1**) [15]. Importantly, ncRNAs also contribute to regulation of both innate and adaptive immune responses via several complementary (and often contradictory) mechanisms (reviewed in [16]). For example, *Lnc13* harbors a single nucleotide polymorphism, which contributes to the onset of type 1 diabetes by indirectly stabilizing STAT1 mRNA and thus prolonging pro-inflammatory signaling [17]. We hypothesized that the changes in lncRNA expression due to co-treatments with TX or CQ also affect how MIAMI cells respond to IFNγ. To test this hypothesis, we examined changes in the expression of lncRNAs in MIAMI cells exposed to IFNγ with or without co-treatment with autophagy modulators TX or CQ to determine which lncRNAs may play a role in MIAMI cells’ response to inflammation.

## Methods

### RNA sequencing

Identities of various differentially regulated RNA species were determined using RNAseq data from our previous study where we reported detailed protocol for cell growth and maintenance, treatments, and data processing [7]. Briefly, MIAMI cells (n = 3) were treated with IFNγ (500 units), TX (5 μM), CQ (10 μM), IFNγ+TX, or IFNγ+TX; total RNA was isolated using RNAzol according to the manufacturer’s instructions (Molecular Research Center). Integrity and concentration of RNA was assessed by Agilent TapeStation 4200 (Agilent Technologies). Total RNA was subjected to 50 million pair-end directional sequencing to a minimum read length of 150 nucleotides using Illumina NextSeq 500 [7]. The raw reads from the 18 samples were used for quality filtering. Clean reads were obtained by removal of reads containing adapter, ploy-N and low quality reads from raw data. Quality control assessment was done using Illumina RNAseq pipeline (RIN number was >9 for all samples) and raw reads were mapped to the reference human genome (the most recent build GRCh38) using GSNAP [18], HISAT2 [19], and STAR aligners [20]. Mapped reads obtained from GSNAP and HISAT2 aligners were counted using HTSeq software [21]. STAR aligner was run with the option “—quantMode GeneCounts”. Raw counts were subsequently imported into Bioconductor/R package DESeq2 (http://www.bioconductor.org/packages/release/bioc/html/ DESeq2.html), normalized and tested for differential gene expression for each aligner. RNA species that were expressed differentially based on the criteria of False Discovery Rate < 5% and Fold Change more than 2.0 in either direction (using at least two different aligners) were examined for their role in inflammation as described in the next section. Data analysis was conducted by reviewing long non-coding RNA species that experienced the most significant changes (>2 fold) compared to untreated cells. Heatmaps of the selected RNA species were created with Heatmapper using the average linkage clustering method and Euclidean distance measuring method applied to rows (http://www1.heatmapper.ca/). The raw RNA sequencing data have been deposited in Gene Expression Omnibus (GEO) at NCBI (https://www.ncbi.nlm.nih.gov/geo/) under the accession number GSE197302.

### Selection of lncRNAs with roles in inflammation

Gene databases http://www.Genecards.org and http://www.ensemble.org combined with the published data from MEDLINE database were utilized to select the RNA species with a potential role in cellular response to inflammation. This role was determined based on any of the following key words incorporated in literature searches: inflammation, NF-κB, immune cells, cytokines, leukocytes, lymphocytes, adaptive immunity, innate immunity, reactive oxygen species. RNA species were also selected based on additional criteria: addition of TX and/or CQ should alter their expression by at least 33% in either direction compared to cells treated with IFNγ. Identified lncRNA, pseudogenes, and antisense RNAs with a potential to contribute to MIAMI cells’ response to inflammation were also analyzed for neighboring genes of interest related to inflammation or inflammatory processes. RNA molecules of interest were sorted by Ensemble number and NCBI Gene ID number. Gene neighbors were then identified using the NCBI database (S1 File). NCBI defines gene neighbors as overlapping genes and the two nearest non-overlapping genes on either side [22]. Each gene neighbor was analyzed for related publications and general gene information to identify any relationship with inflammation. These genes were also searched in the Innate Database, https://www.innatedb.com, a database of genes, proteins, their interactions, and the signaling responses involved in the mammalian innate immunity [23], to confirm each genes’ relation to the inflammatory response.

## Results

**Fig 2:**
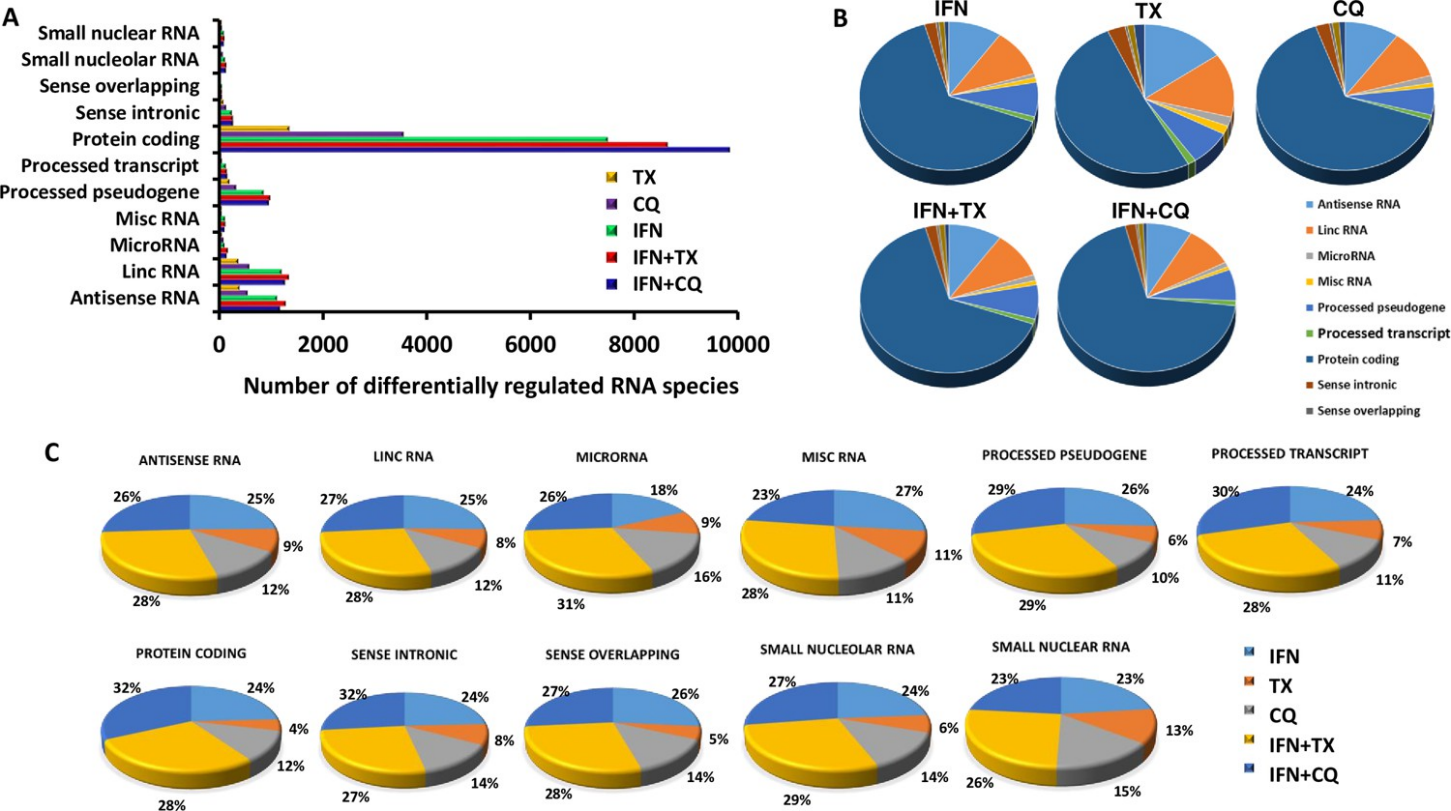
Quantification of differentially regulated ncRNAs using RNA sequencing data. A. Absolute numbers of differentially regulated ncRNAs per treatment; B. Pie charts indicating the percentages of various ncRNAs whose expression changes due to treatments; C. Pie charts indicating changes in percentages of indicated ncRNA types due to treatments.

**Fig 3:**
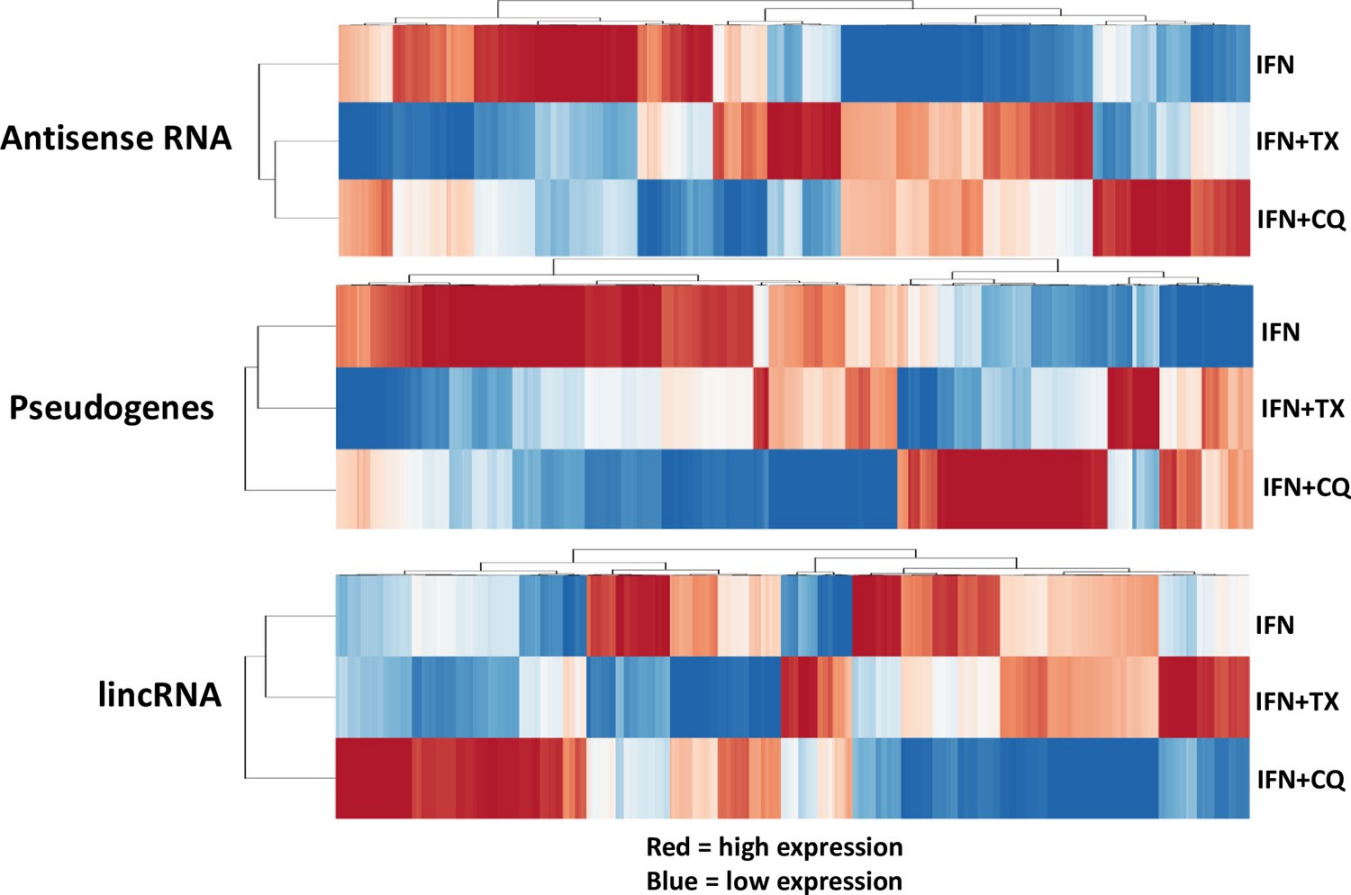
Heatmaps illustrating changes in antisense RNA, pseudogenes, and lincRNA due to MIAMI cells’ exposure to interferon gamma (IFNγ) alone or in combination with chloroquine (CQ) or Tamoxifen (TX).

Utilizing the RNAseq data, total counts for all RNA species were evaluated based on their known roles in various biological processes (**Table 1** and **Fig 2**). It was found that the majority of the differentially regulated RNAs (drRNAs) belong to protein coding RNA, whereas the next three categories of RNAs with a high number of drRNAs were lincRNAs, pseudogenes, and antisense RNAs. Heat maps showing all differentially regulated lncRNAs selected based on the criteria above are depicted on **Fig 3**.

**Table 1:** Total number of differentially regulated RNA species (left column) resulting from indicated treatments (top row).

ncRNA	TX	CQ	IFNγ	IFNγ+TX	IFNγ+CQ
Antisense RNA	398	547	1118	1282	1173
Linc RNA	367	586	1207	1346	1263
MicroRNA	53	90	102	173	147
Misc RNA	49	52	120	125	102
Processed pseudogene	201	331	860	984	958
Processed transcript	39	59	130	152	159
Protein coding	1357	3558	7493	8649	9850
Sense intronic	86	140	245	271	270
Sense overlapping	11	30	57	60	57
Small nucleolar RNA	32	68	117	141	135
Small nuclear RNA	50	61	93	105	94

### Differentially regulated antisense RNAs associated with inflammatory responses

**Fig 4:**
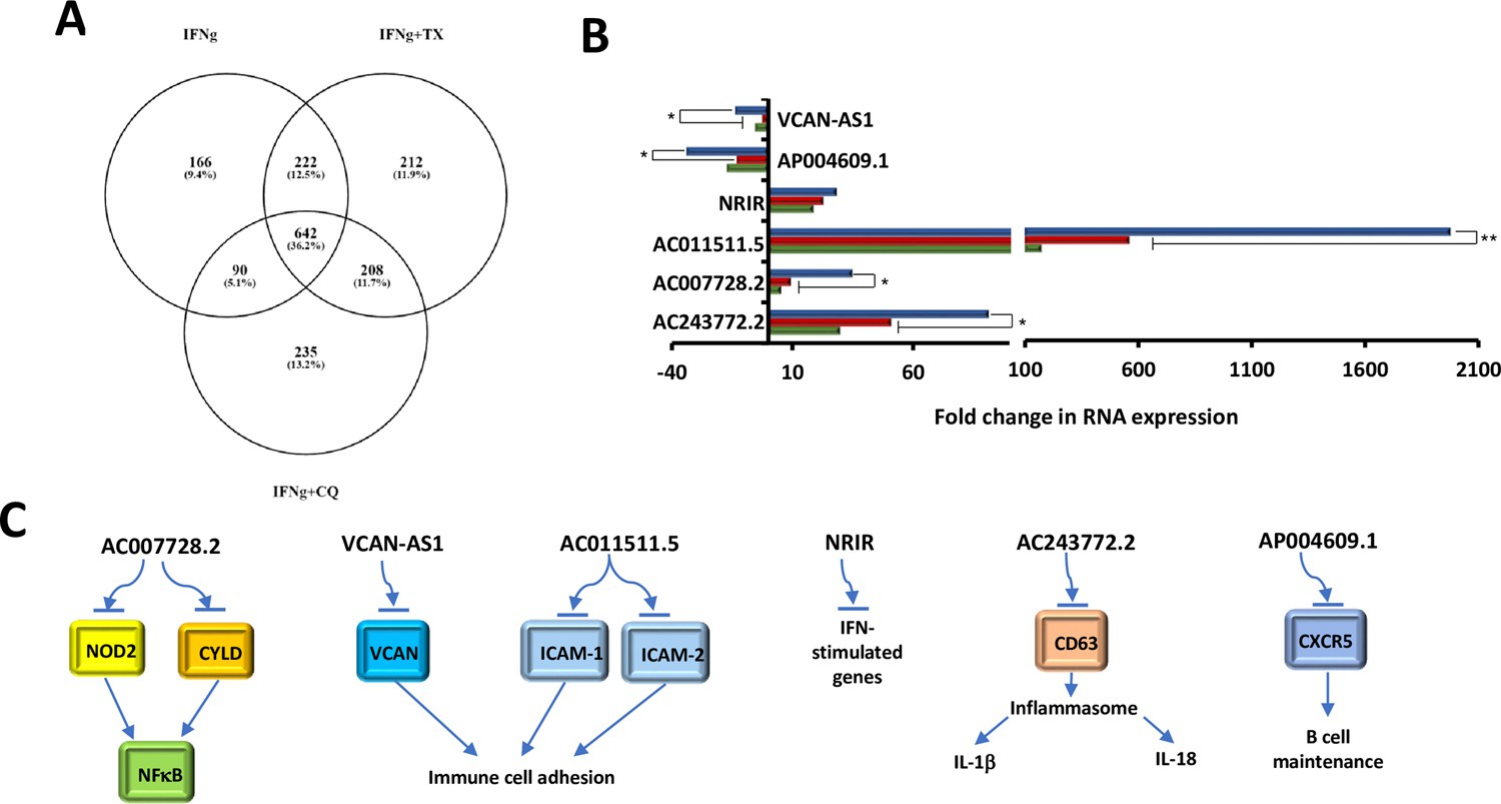
Antisense RNAs involved in MIAMI cells’ response to inflammation. A: Venn diagram showing numbers and percentages of unique and overlapping differentially regulated antisense RNA’s; B: Fold changes in selected antisense RNAs; C: Putative functions of selected RNAs; Green bar IFNγ, red bar IFNγ+TX, blue bar IFNγ+CQ. Values that are significantly different are indicated by bars and asterisks as follows: *, P ≤ 0.05; **, P ≤ 0.01.

Antisense RNAs are single-stranded RNAs that bind to complementary coding strands of mRNA and prevent translation of that transcript [24]. **Fig 1** depicts this interaction and displays the unique function of antisense RNA in expression of genes. Exposure of MIAMI cells to IFNγ resulted in differential regulation of 1120 antisense RNAs; the two drugs caused further changes in 642 antisense RNAs and the putative pro- or anti- inflammatory effects of such changes in MIAMI cells were explored (**Fig 4A**).

Our analysis uncovered two anti-sense RNAs associated with cellular adhesion mechanisms, VCAN-AS1 and AC011511.5. VCAN-AS1 was found to be downregulated 6-fold in response to IFNγ treatment, 3-fold in response to IFNγ+TX, and 14-fold in response to IFNγ+CQ (**Fig 4B**). VCAN-AS1 codes for an antisense RNA to the VCAN gene responsible for the production of the extracellular matrix (ECM) proteoglycan versican by stromal cells and leukocytes [18]. Versican binds to integrin and non-integrin receptors, as well as components of the ECM to ensure stability of cell-cell contacts and thus may also play a major role in controlling inflammation by interacting with myeloid and lymphoid cells to promote their adhesion and facilitate pro-inflammatory cytokine production [18]. Thus, blocking the incorporation of versican into the ECM inhibits monocyte adhesion and reduces the inflammatory response. Further downregulation of VCAN-AS1 with the addition of CQ could enhances IFNγ-induced pro-inflammatory effect(s) by increasing leukocyte adhesion and cytokine release. On the other hand, AC011511.5 was upregulated 19-fold in response to IFNγ treatment, 23-fold in response to IFNγ+TX, and 32-fold in response to IFNγ+CQ (**Fig 4B**). AC011511.5 functions as an antisense RNA to Intercellular Adhesion Molecules 1 and 4 (ICAM-1 and ICAM-4). As an adhesion protein, ICAM-1 plays a significant role in the inflammatory recruitment of leukocytes in response to IL-1 and TNFα. ICAM-1 is found on endothelial cells and functions as a ligand for LFA-1 found on leukocytes allowing them to bind to endothelial cells and transmigrate into tissues [25]. Increasing the expression of AC011511.5 in MIAMI cells would likely result in enhancement of cellular anti-inflammatory properties (**Fig 4C**).

Additionally, two anti-sense RNAs, AC007728.2 and AC243772.2, were found to regulate inflammation by impacting NFκB pathway. AC007728.2 was upregulated 30-fold in response to IFNγ treatment, 51-fold in response to IFNγ+TX and 92-fold in response to IFNγ+CQ (**Fig 4B**). AC007728.2 is an antisense to nucleotide-binding oligomerization domain-containing protein 2 (NOD2) and Cylindromatosis Lysine 63 Deubiquitinase (CYLD) [19]. NOD2 plays a role in the immune response to intracellular bacterial lipopolysaccharides by triggering activation of MAP kinases and NFκB signaling and mutations in NOD2 have been correlated with increased risk of Crohn’s disease [20]. CYLD is a deubiquitinating enzyme involved in regulation of NFκB signaling [21]. Further increase of AC007728.2 upon addition of TX or CQ likely increases MIAMI cells’ anti-inflammatory capacity by decreasing pro-inflammatory signaling (**Fig 4C**). AC243772.2 was upregulated 5-fold in response to IFNγ treatment, 9-fold in response to IFNγ+TX, and 35-fold in response to IFNγ+CQ (**Fig 4B**). AC243772.2 is an antisense RNA to the high affinity immunoglobulin gamma Fc receptor I (FcγRI/CD64), a gene that has been found to have a positive correlation with inflammation. FcγRI increases the formation of the NLRP3 inflammasome and release of the IL-1β and IL-18 cytokines resulting in the activation of NFκB pathway [22]. Moreover, FcγRI expression on neutrophils was found to be significantly elevated in pediatric-onset Crohn’s disease patients [23]. Thus, further upregulation of AC243772.2 in MIAMI cells with the addition of TX or CQ likely enhances cellular anti-inflammatory properties (**Fig 4C**).

Our data analysis also indicates a potential role for interferon signaling pathway. Negative regulator of interferon response (NRIR) is an antisense RNA that was shown to affect the expression of IFN-stimulated genes and its dysregulation contributes to autoimmune diseases such as systemic sclerosis [25]. While NRIR was upregulated 172-fold in response to IFNγ treatment, co-treatments with TX or CQ further upregulated its expression to 562-fold and 1978-fold, respectively (**Fig 4B**). Further increase of NRIR expression with the addition of TX or CQ likely have an anti-inflammatory effect in MIAMI cells (**Fig 4C**).

AP004609.1, a novel antisense RNA to C-X-C chemokine receptor type 5 (CXCR5), was found to be downregulated 17-fold in response to IFNγ treatment, 13-fold in response to TX+IFNγ, and 34-fold in response to CQ+IFNγ (**Fig 4B**). CXCR5 is a transmembrane receptor for the chemokine C-X-C Motif Chemokine Ligand 13 (CXCL13) [26]. This cytokine plays a large role in the orchestration of secondary lymphoid tissue and lymphoid neogenesis [27]. CXCR5-CXCL13 interaction has been shown to play a role in the maintenance of pathogenic B cells in several autoimmune diseases, including rheumatoid arthritis, Sjögren’s syndrome, Hashimoto thyroiditis, Graves’ disease, multiple sclerosis, and myasthenia gravis [28]. Further decrease in AP004609.1 levels with the addition of CQ could result in increased pro-inflammatory response (**Fig 4C**).

### Differentially regulated pseudogenes associated with inflammatory responses

**Fig 5:**
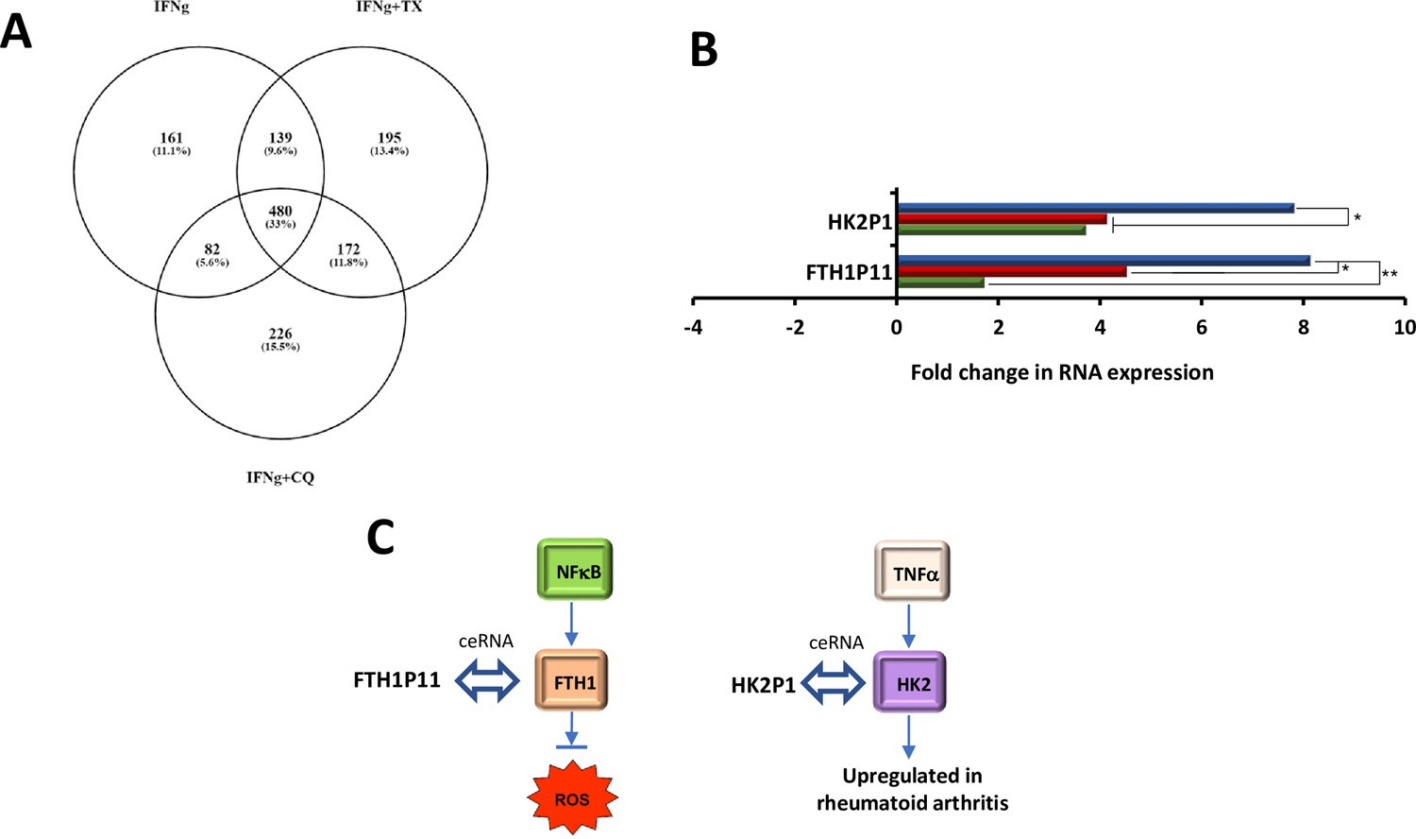
Pseudogenes involved in MIAMI cells’ response to inflammation. A: Venn diagram showing numbers and percentages of unique and overlapping differentially regulated pseudogenes; B: Fold changes in selected pseudogenes; C: Putative functions of selected RNAs; green bar IFNγ, red bar IFNγ+TX, blue bar IFNγ+CQ. Values that are significantly different are indicated by bars and asterisks as follows: *, P ≤ 0.05; **, P ≤ 0.01.

Though most pseudogenes do not possess protein coding potential, many have been found to play a role in the regulation of their parent genes and other genomic loci through a variety of regulatory RNA mechanisms. For example, pseudogenes can modulate gene expression via competing endogenous RNA (ceRNA) activity, transcription into siRNAs and lncRNAs and can modify chromatin architecture [29]. RNAseq yielded a total of 862 differentially regulated pseudogenes upon IFNγ treatment and further found 480 whose expression was changed by addition of TX or CQ (**Fig 5A**).

Our analysis focused on two pseudogenes which met our criteria, Ferritin Heavy Chain 1 Pseudogene 11 (FTH1P11) and Hexokinase 2 Pseudogene 1 (HK2P1), which were shown previously to respond to pro-inflammatory cytokine signaling. Mechanistically, both pseudogenes regulate levels of ceRNAs although via different mechanisms. FTH1P11 was upregulated 2-fold in response to IFNγ treatment, 5-fold in response to IFNγ+TX, and 8-fold in response to IFNγ+CQ (**Fig 5B**). An increase in FTH1P11 increases expression of its parent gene FTH1 which sequesters free iron and mitigates pro-inflammatory ROS production, thus has both antioxidant and anti-inflammatory roles [30]. This regulation may occur through a ceRNA that acts as a molecular sponge for miRNAs that negatively regulate FTH1 gene expression [31]. During inflammation, NFκB upregulates FTH1 and suppresses JNK, which works downstream of TNFα to mediate apoptosis induction [32]. Therefore, co-treatment with either TX or CQ potentially enhance anti-inflammatory cellular response (**Fig 5C**).

HK2P1 was upregulated 4-fold in response to IFNγ, 4-fold in response to IFNγ+TX, and 8-fold in response to IFNγ+CQ treatments (**Fig 5B**). HK2P1 regulates the expression of its parent gene hexokinase 2 (HK2) via a ceRNA that competes with HK2 for the shared miR-6887-3p binding site [33]. This ceRNA competitively inhibits miRNAs that repress HK2, therefore, downregulation of HK2P1 allows for more miR-6887-3p binding to HK2 thus lowering its expression. Interestingly, H2K is upregulated in rheumatoid arthritis (RA), a chronic inflammatory disorder of the joints. Inflammatory signals, including TNFα, have been shown to increase HK2 expression in RA by activating and increasing nuclear localization of its transcriptional regulator, yes-associated protein 1 [34]. Addition of CQ would likely stimulate pro-inflammatory mechanisms that are mediated via HK2 (**Fig 5C**).

### lincRNAs associated with inflammatory responses

**Fig 6:**
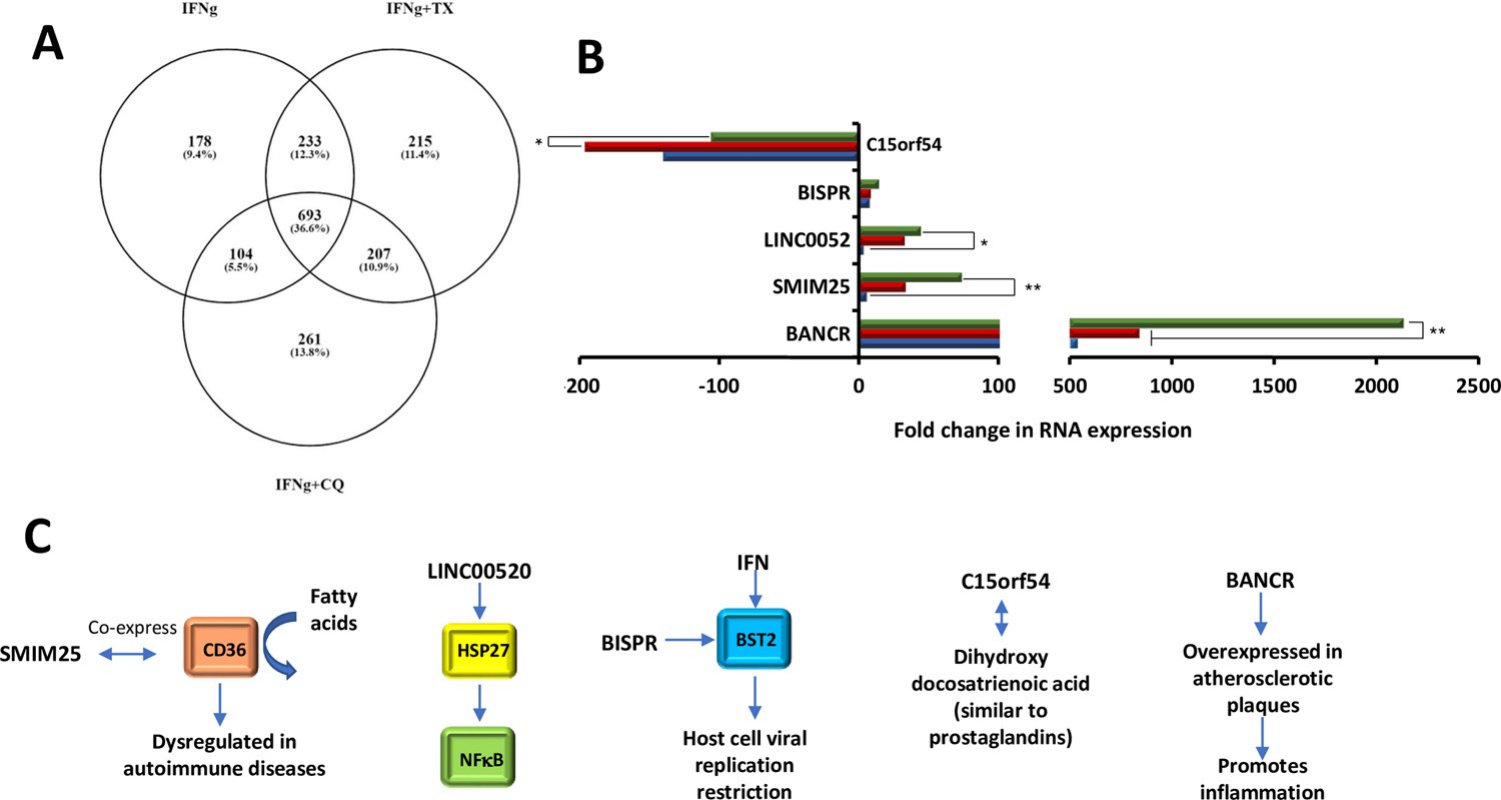
lincRNAs involved in MIAMI cells’ response to inflammation. A: A: Venn diagram showing numbers and percentages of unique and overlapping differentially regulated lincRNAs; B: Fold changes in selected lincRNAs; C: Putative functions of the selected molecules; Green bar IFNγ, Red IFNγ+TX, blue IFNγ+CQ. Values that are significantly different are indicated by bars and asterisks as follows: *, P ≤ 0.05; **, P ≤ 0.01.

Exposure of MIAMI cells to IFNγ resulted in differential regulation of 1208 lincRNAs. Furthermore, 693 lncRNAs were additionally regulated with the addition of TX or CQ and 5 lincRNAs were found to have a potential role in regulating response to inflammation based on the available literature (**Fig 6A**). Our analysis indicated that such differentially regulated lincRNAs are linked to NFκB regulation (LINC00520), lipid metabolism (SMIM25 and BANCR), cell proliferation/apoptosis (BISPR) and dihydroxy-docosatrienoic acid-mediated pathway (C15orf54) as outlined below.

While IFNγ treatment led to an increase of 3.9-fold for LINC00520, co-treatments with TX or CQ led to further upregulation of 32.7-fold and 44.8-fold, respectively (**Fig 6B**). LINC00520 functions as a ceRNA that increases Heat Shock Protein 27 (HSP27) expression [35] resulting in translocation of NFκB protein complex to the nucleus of cells and initiating pro-inflammatory gene expression [36, 37]. Therefore, TX or CQ co-treatments have a potential to further stabilize this transcription factor and stimulate pro-inflammatory signaling (**Fig 6C**).

Similarly, IFNγ treatment led to a 6.0-fold increase for Small Integral Membrane Protein 25 (SMIM25) while addition of TX or CQ led to its further upregulation of 33.0-fold and 74.0-fold, respectively (**Fig 6B**). SMIM25 co-expresses with CD36, a scavenger receptor characterized by high affinity for uptake of long chain fatty acids, which allows for phagocytosis in monocytes [38]. Dysregulation of this gene is seen in several autoimmune diseases of the gastrointestinal tract such as ulcerative colitis and Crohn’s disease [39]. While upregulation of this lincRNA likely has a pro-inflammatory effect in macrophages [38], its effect on MIAMI cells’ immunomodulatory properties is currently unknown (**Fig 6C**).

The levels of oncogene BRAF-activated non-protein coding RNA (BANCR) was increased 538-fold with IFNγ treatment alone and co-treatments with TX or CQ led to its further upregulation of 842-fold and 2134-fold, respectively (**Fig 6B**). BANCR is a lincRNA that facilitates the JNK signaling pathway in vascular smooth muscle cells (VSMC) allowing for VSMC proliferation and migration [40]. Upregulation of BANCR is seen in atherosclerotic plaque and promotes inflammation. BANCR could affect MIAMI cells’ pro-inflammatory response by regulating JNK signaling. Upregulation of BANCR is likely to stimulate both inflammation and cellular proliferation in MIAMI cells (**Fig 6C**).

IFNγ treatment alone increased 8.4-fold BST2 Interferon Stimulated Positive Regulator (BISPR), a regulator of cell proliferation, apoptosis, and antiviral response [41], while co-treatments with TX or CQ led to its further upregulation of 8.4-fold and 14.9-fold, respectively (**Fig 6B**). BISPR is a lincRNA that binds to miR-21-5p, a miRNA that suppresses Bcl-2 expression. miR-21-5p was previously found to promote thyroid papillary carcinoma cell (TPC) viability, propagation, and invasiveness [42]. Studies have shown that BISPR knockout inhibited the proliferation and invasion of TPC cells and helped promote apoptosis. Upregulation of BISPR could promote pro-inflammatory environment as increased cell proliferation leads to an increase in reactive oxygen species within cells and tissues (**Fig 6C**).

Chromosome 15 putative open reading frame 54 (C15orf54) is a lincRNA that has a single nucleotide polymorphism, SNP 17691453, associated with the metabolite dihydroxy-docosatrienoic acid, a biomarker found in heart failure [43]. IFNγ treatment led to a decrease of 140.2-fold for C15orf54, co-treatments with TX or CQ led to its further downregulation of 196.1-fold and 105.9-fold, respectively (**Fig 6B**). Dihydroxy-docosatrienoic acid is produced by the Cytochrome P450 pathway and exerts similar effects to prostaglandins produced by the cyclooxygenase pathway. Downregulation of C15orf54 is likely anti-inflammatory as it would reduce the amount of the pro-inflammatory compound dihydroxy-docosatrienoic acid (**Fig 6C**) [44].

### Identification of neighboring genes

Since lncRNAs may regulate the expression of nearby genes by multiple mechanisms, the neighboring genes for the thirteen lncRNAs identified as potentially involved in modulating MIAMI cells’ response to inflammation were also examined. A total of seventy-four gene neighbors were identified based on the criteria described in Methods section (S1 File), of which four gene neighbors were found to have the potential to regulate the inflammatory process: versican (VCAN), hyaluronan and proteoglycan link protein 1 (HAPLN1), heart and neural crest derivatives expressed 2 (HAND2), and fatty acid binding protein 4 (FABP4). The reported functions of the identified gene neighbors can be found in the **Table 2**. In summary, they were shown to regulate cell adhesion, cytokine expression and pro-inflammatory signaling. This analysis indicate that lncRNAs have the potential to expand their roles by affecting the expression of adjacent genes.

**Table 2:** Identities, ensembl numbers, and functions of neighboring genes that have a potential to be involved in immune response.

lncRNA	Gene Neighbor of Interest	Contribution to Immune function
VCAN-AS1 (ENSG00000249835)	VCAN (ENSG00000038427)	Interacts with immune cells via hyaluronan or via adhesion receptors (CD44, PSGL-1 and TLRs) present on cell surface resulting in activation of NFκB, synthesis and secretion of inflammatory cytokines such as TNFα and IL-6. Also contributes to inflammation regulation by interacting with growth factors and cytokines thereby influencing their bioavailability and activity [55].
VCAN-AS1 (ENSG00000249835)	HAPLN1 (ENSG00000145681)	Induces non-canonical NF-κB activation in multiple myeloma cells [56].
MORF4 (ENSG00000234801)	HAND2 (ENSG00000164107)	Regulates IL15 expression in fibroblasts and fibroblast-derived IL15 is chemotactic for natural killer cells [57].
FTH1P11 (ENSG00000237264)	FABP4 (ENSG00000170323)	Modulates pro-inflammatory cytokine production in adipocytes. Its expression correlates positively with leptin, IL6, TNFR1, and CRP [58].

## Discussion

There is a significant clinical need to enhance anti-inflammatory properties of therapeutic MSCs, a versatile cell-based therapy for diseases where small molecules alone fall short of therapeutic benefits, such as conditions caused by chronic inflammation [45]. In a pro-inflammatory environment, MSCs typically increase expression and secretion of proteins and nucleic acids with anti-inflammatory properties, resulting in creation of an immunomodulatory environment [46]. Unfortunately, the usage of MSCs in clinical practice thus far has been hampered by a need for repeated administration of a high number of cells in the treated subjects resulting in high cost of such therapies [47]. By using a subpopulation of MSCs called MIAMI cells, we evaluated approaches designed to enhance MSCs anti-inflammatory efficacy [7]. In our previous proof-of-principle study, the cells were exposed to IFNγ in the presence of autophagy stimulator TX or autophagy inhibitor CQ. The two autophagy modulators were chosen because autophagy has been shown to play a role in regulating cellular response to inflammation [48]. We found that co-treatment with either drug changed levels of numerous mRNAs and proteins known to play roles in MSCs immunoregulatory properties, resulting in enhancement of cellular ability to decrease CD4 T cell activation and proliferation [7]. We also found that the co-treatments altered the levels of several microRNAs which target mRNAs of genes known to be involved in immunoregulation. Here, we report on additional inflammation-related molecular mechanisms that could influence MIAMI cells’ gene expression in a pro-inflammatory milieu and we report lncRNAs candidates that could be associated with (and potentially predictive of) the anti-inflammatory properties of MSCs.

To evaluate these additional molecular candidates, we focused our analysis on the three lncRNA types, pseudogenes, antisense RNA, and lincRNAs whose expression was additionally altered with co-treatments with TX or CQ. These two drugs were shown to both change the levels of immunomodulatory genes in MIAMI cells and increase cells’ ability to downregulate CD4 T cell activation. This was surprising as we expected that the effect of autophagy stimulation vs. inhibition on MIAMI cells’ immunomodulatory capacity will be in opposite for the two drugs. To explain this observation, we next evaluated additional regulatory mechanisms which are shown to be associated with inflammation signaling and processes.

Most lncRNAs such as pseudogenes, antisense RNA and lincRNAs do not possess protein coding potential [49]. Nevertheless, these RNA species are important for the regulation of gene expression through a variety of additional mechanisms (**Fig 1**) [50]. In the context of MSC homeostasis and multi-differentiation, accumulating evidence supports the role of lncRNAs as regulators of such processes via diverse mechanisms. For example, lncRNA H19 is a key regulator in the multi-lineage commitment of MSCs: it promotes MSC osteogenic differentiation by acting as a ceRNA which inhibits the expression of miR-22 and miR-141 and it also promotes MSC osteogenesis by harvesting miR-138 resulting in the activation of FAK pathway [51, 52]. Moreover, lncRNAs are also known to play a regulatory role in both innate and adaptive immunity as well as in cellular response to infection and inflammation (reviewed in [16]).

Unfortunately, much less is known regarding the role of lncRNAs in MSCs therapeutic efficacy. Importantly, in addition to mediating MSCs’ response to inflammation via intracellular mechanisms, lncRNAs may also be delivered via extracellular vesicles (such as exosomes) to target cells, and thus could contribute to the regulation of target cells’ inflammatory state [53, 54]. Therefore, we report here the potential roles for differentially regulated lncRNAs in response to IFNγ treatment by evaluating the known functions of lncRNAs based on the criteria outlined in the Methods section. Our results indicate that the biological processes and pathways that may be altered due to TX or CQ co-treatments include NFκB signaling pathway, IFN-stimulated genes, ROS, myeloid interactions with immunoglobulin, leukocyte recruitment, cell adhesion and chemokine responses (**Figs 4–6**). While some of the lncRNAs profiled here had a direct role in mediating inflammation (NRIR, AC243772.2, and LINC00520 to mention a few), others (SMIM25, FTH1P11, VCAN-AS1, and AC011511.5) have been linked to the inflammatory process indirectly. These observations suggests that changes in expression of lncRNAs have a potential to alter MSCs’ response to inflammation via lncRNAs’ effects on multiple direct and indirect inflammatory pathways and molecular mechanisms. Our analysis also uncovered that lncRNAs examined here are linked to the regulation of both pro- and anti-inflammatory mechanisms; moreover, addition of TX or CQ altered the expression of molecules in the same direction. This is somewhat unexpected and could be indicative of why both drugs increased MIAMI cells’ immunomodulatory capacity to a similar extent and in the same direction [7]. It is likely that the measured functional change in MIAMI cells’ ability to downregulate CD4 T cell activation results from alterations in the levels of multiple pro- and anti-inflammatory molecules and pathways which includes a number of proteins and RNA species.

## Conclusion

Altogether, the present study profiled the differentially expressed of lncRNAs in MIAMI cells that were exposed to IFNγ in the presence of TX or CQ; the study evaluated putative regulatory roles and associated pathways for multiple lncRNA. A number of pathways that include both pro-and anti-inflammatory ones were examined, including those mediated via cytokine signaling and several that are linked to autoimmune responses. Future exploration and validation of lncRNAs expression should be used to define lncRNAs’ roles in MIAMI cell-mediated immunomodulation using functional assays. Additionally, because we only evaluated lncRNAs with previously studied functions based on the available literature, the mechanistic role of other differentially regulated lncRNAs with currently unknown functions should be considered in further investigations.

## Supporting information

S1 File LincRNAs involved in MIAMI cells’ response to inflammation (top paragraph) and their gene neighbors (bottom paragraph). (DOCX)

S1 Data Excel sheet containing average values of all differentially regulated lincRNAs and their expression values (vs untreated) upon exposures to IFNγ, IFNγ+CQ and IFNγ+TX. (XLSX)

S2 Data Excel sheet containing average values of all differentially regulated antisense RNAs and their expression values (vs untreated) upon exposures to IFNγ, IFNγ+CQ and IFNγ+TX. (XLSX)

S3 Data Excel sheet containing average values of all differentially regulated pseudogenes and their expression values (vs untreated) upon exposures to IFNγ, IFNγ+CQ and IFNγ+TX. (XLSX)

## References

[1] Pittenger MF, Discher DE, Peault BM, Phinney DG, Hare JM, Caplan AI. Mesenchymal stem cell perspective: cell biology to clinical progress. NPJ Regen Med. 2019;4:22. Epub 2019/12/10. doi: 10.1038/s41536-019-0083-6 ; PubMed Central PMCID: PMC6889290. 31815001 PMC6889290

[2] Weiss ARR, Dahlke MH. Immunomodulation by Mesenchymal Stem Cells (MSCs): Mechanisms of Action of Living, Apoptotic, and Dead MSCs. Front Immunol. 2019;10:1191. Epub 2019/06/20. doi: 10.3389/fimmu.2019.01191 ; PubMed Central PMCID: PMC6557979. 31214172 PMC6557979

[3] Mosanya CH, Isaacs JD. Tolerising cellular therapies: what is their promise for autoimmune disease? Ann Rheum Dis. 2019;78(3):297–310. Epub 2018/11/06. doi: 10.1136/annrheumdis-2018-214024 ; PubMed Central PMCID: PMC6390030. 30389690 PMC6390030

[4] Neri S, Borzi RM. Molecular Mechanisms Contributing to Mesenchymal Stromal Cell Aging. Biomolecules. 2020;10(2). Epub 2020/02/27. doi: 10.3390/biom10020340 ; PubMed Central PMCID: PMC7072652. 32098040 PMC7072652

[5] Li Y, Wu Q, Wang Y, Li L, Bu H, Bao J. Senescence of mesenchymal stem cells (Review). Int J Mol Med. 2017;39(4):775–82. Epub 2017/03/16. doi: 10.3892/ijmm.2017.2912 . 28290609

[6] Ceccarelli S, Pontecorvi P, Anastasiadou E, Napoli C, Marchese C. Immunomodulatory Effect of Adipose-Derived Stem Cells: The Cutting Edge of Clinical Application. Front Cell Dev Biol. 2020;8:236. Epub 2020/05/05. doi: 10.3389/fcell.2020.00236 ; PubMed Central PMCID: PMC7180192. 32363193 PMC7180192

[7] Rossi F, Noren H, Sarria L, Schiller PC, Nathanson L, Beljanski V. Combination therapies enhance immunoregulatory properties of MIAMI cells. Stem Cell Res Ther. 2019;10(1):395. Epub 2019/12/20. doi: 10.1186/s13287-019-1515-3 ; PubMed Central PMCID: PMC6921447. 31852519 PMC6921447

[8] Karantalis V, Suncion-Loescher VY, Bagno L, Golpanian S, Wolf A, Sanina C, et al. Synergistic Effects of Combined Cell Therapy for Chronic Ischemic Cardiomyopathy. J Am Coll Cardiol. 2015;66(18):1990–9. Epub 2015/10/31. doi: 10.1016/j.jacc.2015.08.879 ; PubMed Central PMCID: PMC4628729. 26516002 PMC4628729

[9] Jiang GM, Tan Y, Wang H, Peng L, Chen HT, Meng XJ, et al. The relationship between autophagy and the immune system and its applications for tumor immunotherapy. Mol Cancer. 2019;18(1):17. Epub 2019/01/27. doi: 10.1186/s12943-019-0944-z ; PubMed Central PMCID: PMC6345046. 30678689 PMC6345046

[10] Yin Z, Pascual C, Klionsky DJ. Autophagy: machinery and regulation. Microb Cell. 2016;3(12):588–96. Epub 2017/03/31. doi: 10.15698/mic2016.12.546 ; PubMed Central PMCID: PMC5348978. 28357331 PMC5348978

[11] Kaikkonen MU, Lam MT, Glass CK. Non-coding RNAs as regulators of gene expression and epigenetics. Cardiovasc Res. 2011;90(3):430–40. Epub 2011/05/12. doi: 10.1093/cvr/cvr097 ; PubMed Central PMCID: PMC3096308. 21558279 PMC3096308

[12] Gomes AQ, Nolasco S, Soares H. Non-coding RNAs: multi-tasking molecules in the cell. Int J Mol Sci. 2013;14(8):16010–39. Epub 2013/08/06. doi: 10.3390/ijms140816010 ; PubMed Central PMCID: PMC3759897. 23912238 PMC3759897

[13] Fernandes JCR, Acuna SM, Aoki JI, Floeter-Winter LM, Muxel SM. Long Non-Coding RNAs in the Regulation of Gene Expression: Physiology and Disease. Noncoding RNA. 2019;5(1). Epub 2019/02/20. doi: 10.3390/ncrna5010017 ; PubMed Central PMCID: PMC6468922. 30781588 PMC6468922

[14] Han Y, Li X, Zhang Y, Han Y, Chang F, Ding J. Mesenchymal Stem Cells for Regenerative Medicine. Cells. 2019;8(8). Epub 2019/08/16. doi: 10.3390/cells8080886 ; PubMed Central PMCID: PMC6721852. 31412678 PMC6721852

[15] Long Y, Wang X, Youmans DT, Cech TR. How do lncRNAs regulate transcription? Sci Adv. 2017;3(9):eaao2110. Epub 2017/09/30. doi: 10.1126/sciadv.aao2110 ; PubMed Central PMCID: PMC5617379. 28959731 PMC5617379

[16] Chen J, Ao L, Yang J. Long non-coding RNAs in diseases related to inflammation and immunity. Ann Transl Med. 2019;7(18):494. Epub 2019/11/09. doi: 10.21037/atm.2019.08.37 ; PubMed Central PMCID: PMC6803193. 31700930 PMC6803193

[17] Gonzalez-Moro I, Olazagoitia-Garmendia A, Colli ML, Cobo-Vuilleumier N, Postler TS, Marselli L, et al. The T1D-associated lncRNA Lnc13 modulates human pancreatic beta cell inflammation by allele-specific stabilization of STAT1 mRNA. Proc Natl Acad Sci U S A. 2020;117(16):9022–31. Epub 2020/04/15. doi: 10.1073/pnas.1914353117 ; PubMed Central PMCID: PMC7183221. 32284404 PMC7183221

[18] Wu TD, Nacu S. Fast and SNP-tolerant detection of complex variants and splicing in short reads. Bioinformatics. 2010;26(7):873–81. Epub 2010/02/12. doi: 10.1093/bioinformatics/btq057 ; PubMed Central PMCID: PMC2844994. 20147302 PMC2844994

[19] Kim D, Langmead B, Salzberg SL. HISAT: a fast spliced aligner with low memory requirements. Nat Methods. 2015;12(4):357–60. Epub 2015/03/10. doi: 10.1038/nmeth.3317 ; PubMed Central PMCID: PMC4655817. 25751142 PMC4655817

[20] Dobin A, Davis CA, Schlesinger F, Drenkow J, Zaleski C, Jha S, et al. STAR: ultrafast universal RNA-seq aligner. Bioinformatics. 2013;29(1):15–21. Epub 2012/10/30. doi: 10.1093/bioinformatics/bts635 ; PubMed Central PMCID: PMC3530905. 23104886 PMC3530905

[21] Anders S, Pyl PT, Huber W. HTSeq—a Python framework to work with high-throughput sequencing data. Bioinformatics. 2015;31(2):166–9. Epub 2014/09/28. doi: 10.1093/bioinformatics/btu638 ; PubMed Central PMCID: PMC4287950. 25260700 PMC4287950

[22] Fishilevich S, Nudel R, Rappaport N, Hadar R, Plaschkes I, Iny Stein T, et al. GeneHancer: genome-wide integration of enhancers and target genes in GeneCards. Database (Oxford). 2017;2017. Epub 2017/06/13. doi: 10.1093/database/bax028 ; PubMed Central PMCID: PMC5467550. 28605766 PMC5467550

[23] Breuer K, Foroushani AK, Laird MR, Chen C, Sribnaia A, Lo R, et al. InnateDB: systems biology of innate immunity and beyond—recent updates and continuing curation. Nucleic Acids Res. 2013;41(Database issue):D1228–33. Epub 2012/11/28. doi: 10.1093/nar/gks1147 ; PubMed Central PMCID: PMC3531080. 23180781 PMC3531080

[24] Brantl S. Antisense-RNA regulation and RNA interference. Biochim Biophys Acta. 2002;1575(1–3):15–25. Epub 2002/05/22. doi: 10.1016/s0167-4781(02)00280-4 . 12020814

[25] Mariotti B, Servaas NH, Rossato M, Tamassia N, Cassatella MA, Cossu M, et al. The Long Non-coding RNA NRIR Drives IFN-Response in Monocytes: Implication for Systemic Sclerosis. Front Immunol. 2019;10:100. Epub 2019/02/26. doi: 10.3389/fimmu.2019.00100 ; PubMed Central PMCID: PMC6371048. 30804934 PMC6371048

[26] Kazanietz MG, Durando M, Cooke M. CXCL13 and Its Receptor CXCR5 in Cancer: Inflammation, Immune Response, and Beyond. Front Endocrinol (Lausanne). 2019;10:471. Epub 2019/07/30. doi: 10.3389/fendo.2019.00471 ; PubMed Central PMCID: PMC6639976. 31354634 PMC6639976

[27] Carlsen HS, Baekkevold ES, Morton HC, Haraldsen G, Brandtzaeg P. Monocyte-like and mature macrophages produce CXCL13 (B cell-attracting chemokine 1) in inflammatory lesions with lymphoid neogenesis. Blood. 2004;104(10):3021–7. Epub 2004/07/31. doi: 10.1182/blood-2004-02-0701 . 15284119

[28] Finch DK, Ettinger R, Karnell JL, Herbst R, Sleeman MA. Effects of CXCL13 inhibition on lymphoid follicles in models of autoimmune disease. Eur J Clin Invest. 2013;43(5):501–9. Epub 2013/03/23. doi: 10.1111/eci.12063 . 23517338

[29] Cheetham SW, Faulkner GJ, Dinger ME. Overcoming challenges and dogmas to understand the functions of pseudogenes. Nat Rev Genet. 2020;21(3):191–201. Epub 2019/12/19. doi: 10.1038/s41576-019-0196-1 . 31848477

[30] Tsuji Y, Ayaki H, Whitman SP, Morrow CS, Torti SV, Torti FM. Coordinate transcriptional and translational regulation of ferritin in response to oxidative stress. Mol Cell Biol. 2000;20(16):5818–27. Epub 2000/07/27. doi: 10.1128/MCB.20.16.5818-5827.2000 ; PubMed Central PMCID: PMC86059. 10913165 PMC86059

[31] Chan JJ, Kwok ZH, Chew XH, Zhang B, Liu C, Soong TW, et al. A FTH1 gene:pseudogene:microRNA network regulates tumorigenesis in prostate cancer. Nucleic Acids Res. 2018;46(4):1998–2011. Epub 2017/12/15. doi: 10.1093/nar/gkx1248 ; PubMed Central PMCID: PMC5829750. 29240947 PMC5829750

[32] Pham CG, Bubici C, Zazzeroni F, Papa S, Jones J, Alvarez K, et al. Ferritin heavy chain upregulation by NF-kappaB inhibits TNFalpha-induced apoptosis by suppressing reactive oxygen species. Cell. 2004;119(4):529–42. Epub 2004/11/13. doi: 10.1016/j.cell.2004.10.017 . 15537542

[33] Lv H, Tong J, Yang J, Lv S, Li WP, Zhang C, et al. Dysregulated Pseudogene HK2P1 May Contribute to Preeclampsia as a Competing Endogenous RNA for Hexokinase 2 by Impairing Decidualization. Hypertension. 2018;71(4):648–58. Epub 2018/02/15. doi: 10.1161/HYPERTENSIONAHA.117.10084 . 29440331

[34] Gao Y, Yang Y, Yuan F, Huang J, Xu W, Mao B, et al. TNFalpha-YAP/p65-HK2 axis mediates breast cancer cell migration. Oncogenesis. 2017;6(9):e383. Epub 2017/09/26. doi: 10.1038/oncsis.2017.83 ; PubMed Central PMCID: PMC5623908. 28945218 PMC5623908

[35] Jin XH, Hong YG, Li P, Hao LQ, Chen M. Long noncoding RNA LINC00520 accelerates the progression of colorectal cancer by serving as a competing endogenous RNA of microRNA-577 to increase HSP27 expression. Hum Cell. 2020;33(3):683–94. Epub 2020/03/09. doi: 10.1007/s13577-020-00336-8 . 32146708

[36] Ferns G, Shams S, Shafi S. Heat shock protein 27: its potential role in vascular disease. Int J Exp Pathol. 2006;87(4):253–74. Epub 2006/08/01. doi: 10.1111/j.1365-2613.2006.00484.x ; PubMed Central PMCID: PMC2517372. 16875491 PMC2517372

[37] Salari S, Seibert T, Chen YX, Hu T, Shi C, Zhao X, et al. Extracellular HSP27 acts as a signaling molecule to activate NF-kappaB in macrophages. Cell Stress Chaperones. 2013;18(1):53–63. Epub 2012/08/02. doi: 10.1007/s12192-012-0356-0 ; PubMed Central PMCID: PMC3508120. 22851137 PMC3508120

[38] Hung J, Scanlon JP, Mahmoud AD, Rodor J, Ballantyne M, Fontaine MAC, et al. Novel Plaque Enriched Long Noncoding RNA in Atherosclerotic Macrophage Regulation (PELATON). Arterioscler Thromb Vasc Biol. 2020;40(3):697–713. Epub 2019/12/13. doi: 10.1161/ATVBAHA.119.313430 ; PubMed Central PMCID: PMC7043732. 31826651 PMC7043732

[39] Yarani R, Mirza AH, Kaur S, Pociot F. The emerging role of lncRNAs in inflammatory bowel disease. Exp Mol Med. 2018;50(12):1–14. Epub 2018/12/14. doi: 10.1038/s12276-018-0188-9 ; PubMed Central PMCID: PMC6283835. 30523244 PMC6283835

[40] Li H, Liu X, Zhang L, Li X. LncRNA BANCR facilitates vascular smooth muscle cell proliferation and migration through JNK pathway. Oncotarget. 2017;8(70):114568–75. Epub 2018/02/01. doi: 10.18632/oncotarget.21603 ; PubMed Central PMCID: PMC5777714. 29383102 PMC5777714

[41] Barriocanal M, Carnero E, Segura V, Fortes P. Long Non-Coding RNA BST2/BISPR is Induced by IFN and Regulates the Expression of the Antiviral Factor Tetherin. Front Immunol. 2014;5:655. Epub 2015/01/27. doi: 10.3389/fimmu.2014.00655 ; PubMed Central PMCID: PMC4288319. 25620967 PMC4288319

[42] Zhang H, Cai Y, Zheng L, Zhang Z, Lin X, Jiang N. LncRNA BISPR promotes the progression of thyroid papillary carcinoma by regulating miR-21-5p. Int J Immunopathol Pharmacol. 2018;32:2058738418772652. Epub 2018/06/02. doi: 10.1177/2058738418772652 ; PubMed Central PMCID: PMC5985546. 29856242 PMC5985546

[43] Yu B, Zheng Y, Alexander D, Manolio TA, Alonso A, Nettleton JA, et al. Genome-wide association study of a heart failure related metabolomic profile among African Americans in the Atherosclerosis Risk in Communities (ARIC) study. Genet Epidemiol. 2013;37(8):840–5. Epub 2013/08/13. doi: 10.1002/gepi.21752 ; PubMed Central PMCID: PMC4079107. 23934736 PMC4079107

[44] Gabbs M, Leng S, Devassy JG, Monirujjaman M, Aukema HM. Advances in Our Understanding of Oxylipins Derived from Dietary PUFAs. Adv Nutr. 2015;6(5):513–40. Epub 2015/09/17. doi: 10.3945/an.114.007732 ; PubMed Central PMCID: PMC4561827. 26374175 PMC4561827

[45] Levy O, Kuai R, Siren EMJ, Bhere D, Milton Y, Nissar N, et al. Shattering barriers toward clinically meaningful MSC therapies. Sci Adv. 2020;6(30):eaba6884. Epub 2020/08/25. doi: 10.1126/sciadv.aba6884 ; PubMed Central PMCID: PMC7439491. 32832666 PMC7439491

[46] Song N, Scholtemeijer M, Shah K. Mesenchymal Stem Cell Immunomodulation: Mechanisms and Therapeutic Potential. Trends Pharmacol Sci. 2020;41(9):653–64. Epub 2020/07/28. doi: 10.1016/j.tips.2020.06.009 ; PubMed Central PMCID: PMC7751844. 32709406 PMC7751844

[47] Zhou T, Yuan Z, Weng J, Pei D, Du X, He C, et al. Challenges and advances in clinical applications of mesenchymal stromal cells. J Hematol Oncol. 2021;14(1):24. Epub 2021/02/14. doi: 10.1186/s13045-021-01037-x ; PubMed Central PMCID: PMC7880217. 33579329 PMC7880217

[48] Netea-Maier RT, Plantinga TS, van de Veerdonk FL, Smit JW, Netea MG. Modulation of inflammation by autophagy: Consequences for human disease. Autophagy. 2016;12(2):245–60. Epub 2015/07/30. doi: 10.1080/15548627.2015.1071759 ; PubMed Central PMCID: PMC4836004. 26222012 PMC4836004

[49] Ma L, Bajic VB, Zhang Z. On the classification of long non-coding RNAs. RNA Biol. 2013;10(6):925–33. Epub 2013/05/23. doi: 10.4161/rna.24604 ; PubMed Central PMCID: PMC4111732. 23696037 PMC4111732

[50] Statello L, Guo CJ, Chen LL, Huarte M. Gene regulation by long non-coding RNAs and its biological functions. Nat Rev Mol Cell Biol. 2021;22(2):96–118. Epub 2020/12/24. doi: 10.1038/s41580-020-00315-9 ; PubMed Central PMCID: PMC7754182. 33353982 PMC7754182

[51] Gong YY, Peng MY, Yin DQ, Yang YF. Long non-coding RNA H19 promotes the osteogenic differentiation of rat ectomesenchymal stem cells via Wnt/beta-catenin signaling pathway. Eur Rev Med Pharmacol Sci. 2018;22(24):8805–13. Epub 2018/12/24. doi: 10.26355/eurrev_201812_16648 . 30575922

[52] Hu W, Ye Y, Zhang W, Wang J, Chen A, Guo F. miR1423p promotes osteoblast differentiation by modulating Wnt signaling. Mol Med Rep. 2013;7(2):689–93. Epub 2012/12/12. doi: 10.3892/mmr.2012.1207 . 23229013

[53] van de Vyver M, Powrie YSL, Smith C. Targeting Stem Cells in Chronic Inflammatory Diseases. Adv Exp Med Biol. 2021;1286:163–81. Epub 2021/03/17. doi: 10.1007/978-3-030-55035-6_12 . 33725353

[54] Mendt M, Rezvani K, Shpall E. Mesenchymal stem cell-derived exosomes for clinical use. Bone Marrow Transplant. 2019;54(Suppl 2):789–92. Epub 2019/08/23. doi: 10.1038/s41409-019-0616-z . 31431712

[55] Wight TN, Kang I, Evanko SP, Harten IA, Chang MY, Pearce OMT, et al. Versican-A Critical Extracellular Matrix Regulator of Immunity and Inflammation. Front Immunol. 2020;11:512. Epub 2020/04/09. doi: 10.3389/fimmu.2020.00512 ; PubMed Central PMCID: PMC7105702. 32265939 PMC7105702

[56] Huynh M, Pak C, Markovina S, Callander NS, Chng KS, Wuerzberger-Davis SM, et al. Hyaluronan and proteoglycan link protein 1 (HAPLN1) activates bortezomib-resistant NF-kappaB activity and increases drug resistance in multiple myeloma. J Biol Chem. 2018;293(7):2452–65. Epub 2017/12/28. doi: 10.1074/jbc.RA117.000667 ; PubMed Central PMCID: PMC5818187. 29279332 PMC5818187

[57] Marinic M, Mika K, Chigurupati S, Lynch VJ. Evolutionary transcriptomics implicates HAND2 in the origins of implantation and regulation of gestation length. Elife. 2021;10. Epub 2021/02/02. doi: 10.7554/eLife.61257 ; PubMed Central PMCID: PMC7943190. 33522483 PMC7943190

[58] Terra X, Quintero Y, Auguet T, Porras JA, Hernandez M, Sabench F, et al. FABP 4 is associated with inflammatory markers and metabolic syndrome in morbidly obese women. Eur J Endocrinol. 2011;164(4):539–47. Epub 2011/01/25. doi: 10.1530/EJE-10-1195 . 21257725

