# Supplementary material for: Effects of autophagy modulators tamoxifen and chloroquine on the expression profiles of long non-coding RNAs in MIAMI cells exposed to IFNγ
Source: PLoS One. 2022 Apr 21;17(4):e0266179. doi: 10.1371/journal.pone.0266179 (PMC9022845; doi:10.1371/journal.pone.0266179)
Supplement: S1 File — (DOCX) [file pone.0266179.s001.docx]

**LincRNAs involved in MIAMI cells’ response to inflammation (top paragraph) and their gene neighbors (bottom paragraph):**

LincRNAs involved in MIAMI cells’ response to inflammation: LUARIS (ENSG00000231638), VCAN-AS1 (ENSG00000249835), NRIR (ENSG00000225964), MORF4 (ENSG00000234801), RPL3AP25 (ENSG00000136149), HK2P1 (ENSG00000228612), FTH1P11 (ENSG00000237264), C15orf41 (ENSG00000186073), BISPR (ENSG00000282851), LINC0052 (ENSG00000258791), SMIM25 (ENSG00000224397), BANCR (ENSG00000278910), NOD2 (ENSG00000167207), CYLD (ENSG00000083799), FTH1 (ENSG00000167996)

Gene Neighbors Identified: LOC111591501 (NCBI Gene ID 111591501), LOC102724903, (NCBI Gene ID 102724903), RPL18AP10 (NCBI Gene ID 100271285), COA1(NCBI Gene ID 55744), HECW1 (NCBI Gene ID 23072), STK17A (NCBI Gene ID 9263), LOC107986431 (NCBI Gene ID 107986431), RNU6-620P (NCBI Gene ID 106481377), FTH1P9 (NCBI Gene ID106478936), VCAN (NCBI Gene ID 1462), HAPLN1 (NCBI Gene ID 1404), LOC105373404 (NCBI Gene ID 105373404), GRASLND (NCBI Gene ID 386597), CMPK2 (NCBI Gene ID 129607), RSAD2 (NCBI Gene ID 91543), RNF144A (NCBI Gene ID 9781), LOC107986203 (NCBI Gene ID 107986203), LOC101928409 (NCBI Gene ID 101928409), RANP6 (NCBI Gene ID 100128266), HAND2-AS1 (NCBI Gene ID 79804), SCRG1 (NCBI Gene ID 11341), HAND2 (NCBI Gene ID 9464), LOC105370213 (NCBI Gene ID 105370213), MIR5007 (NCBI Gene ID 100846996), LINC00458 (NCBI Gene ID 100507428), HNRNPH3P1 (NCBI Gene ID 106480233), LOC105373283 (NCBI Gene ID 105373283), PSMA1P1 (NCBI Gene ID 100422286), WBP11P3 (NCBI Gene ID 727874), BRWD3 (NCBI Gene ID 254065), LOC101927118 (NCBI Gene ID 101927118), NIPA2P4 (NCBI Gene ID 100421776), FABP12 (NCBI Gene ID 646486), FABP9 (NCBI Gene ID 646480), FABP4 (NCBI Gene ID 2167), LOC110120936 (NCBI Gene ID 110120936), LOC110120858 (NCBI Gene ID 110120858), LOC110120842 (NCBI Gene ID 110120842), TPST2P1 (NCBI Gene ID 100420710), COX6CP4 (NCBI Gene ID 100288892), LOC751603 (NCBI Gene ID 751603), LARP4P (NCBI Gene ID 554354), CSNK1A1P1 (NCBI Gene ID 161635), LOC145845 (NCBI Gene ID 145845), MEIS2 (NCBI Gene ID 4212), LOC112268485 (NCBI Gene ID 112268485), LOC105370511 (NCBI Gene ID 105370511), RPL13AP3 (NCBI Gene ID 645683), KTN1 (NCBI Gene ID 3895), LOC105372657 (NCBI Gene ID 105372657), LOC105372656 (NCBI Gene ID 105372656), LINC01271 (NCBI Gene ID 101927586), LINC01270 (NCBI Gene ID 284751), LOC108510657 (NCBI Gene ID 108510657), LOC108175349 (NCBI Gene ID 108175349), LOC105376074 (NCBI Gene ID 105376074), TJP2 (NCBI Gene ID 9414), FAM189A2 (NCBI Gene ID 9413), FXN (NCBI Gene ID 2395), APBA1 (NCBI Gene ID 320), LOC111365154 (NCBI Gene ID 111365154), LOC107984334 (NCBI Gene ID 107984334), RNU6-1243P (NCBI Gene ID 106481573), LOC399900 (NCBI Gene ID 399900), BEST1 (NCBI Gene ID 7439), RAB3IL1 (NCBI Gene ID 5866), LINC02168 (NCBI Gene ID 105371252), LOC105371251 (NCBI Gene ID 105371251), CYLD-AS1 (NCBI Gene ID 102724907), LOC101927272 (NCBI Gene ID 101927272), MIR3181 (NCBI Gene ID 100422972), SNX20 (NCBI Gene ID 124460), NKD1 (NCBI Gene ID 85407), LOC110120841 (NCBI Gene ID 110120841)
